# Modeling Extracellular Matrix-Cell Interactions in Lung Repair and Chronic Disease

**DOI:** 10.3390/cells10082145

**Published:** 2021-08-20

**Authors:** Tillie Louise Hackett, Emmanuel Twumasi Osei

**Affiliations:** 1 Centre for Heart Lung Innovation, St. Paul’s Hospital, 1081 Burrard Street, Vancouver, BC V6Z 1Y6, Canada; emmanuel.osei@hli.ubc.ca; 2 Department of Anesthesiology, Pharmacology and Therapeutics, University of British Columbia, Vancouver, BC V6T 1Z3, Canada; 3 Department of Biology, University of British Columbia, Okanagan, BC V6T 1Z4, Canada

## Introduction

The lung extracellular matrix (ECM) is a complex and dynamic mixture of fibrous proteins (collagen, elastin), glycoproteins (fibronectin, laminin), glycosaminoglycans (heparin, hyaluronic acid) and proteoglycans (perlecan, versican), that are essential for normal lung development and organ health. In addition to providing structural integrity, the ECM shapes cell behaviour both in health and disease via its molecular composition, stiffness [1,2], and as a reservoir for growth factors and cytokines. The lung anatomy is complex and includes conducting airways, respiratory airways (including alveoli), lymphatics and the pulmonary vasculature, which all require unique ECM environments to support specialized cell survival, proliferation, and differentiation [3]. Within the lung, the ECM is organized into two main structural types: 1) basement membranes, which are thin, dense sheets of specialized, self-assembled ECM proteins that underlie the airway and alveolar epithelium, vascular endothelial cells, airway and vascular smooth muscle, and 2) an interstitial matrix that is formed of a porous ECM that forms a 3-dimensional (3D) lattice around cells within the lamina propria of conducting airways, adventitia of pulmonary vessels and the parenchymal interstitium. These unique lung ECM environments are, in turn, maintained by the surrounding cells through the production of new ECM molecules, ECM turnover via degradation and ECM organization. Each day, the human lung is continuously exposed to inhaled microbes, allergens, particulate matter, and smoke. In response, a continuous process of repair and generation to maintain ECM homeostasis ensues. However, it is not well understood how cells balance the production of new ECM molecules, or degradation and organization of existing ECM during tissue repair or remodeling in disease. Extensive reorganization of the lung ECM is a pathological alteration that leads to functional changes in lung function for several chronic lung diseases including asthma, chronic obstructive pulmonary disease (COPD) and idiopathic pulmonary fibrosis (IPF). This special issue focuses on how different pulmonary disease models are elucidating ECM repair mechanisms in health and chronic lung disease. The reviews and research articles highlighted assess the contribution of cells in modulating the ECM environment through secreted factors (cytokines and growth factor), epigenetic mechanisms of ECM gene regulation and biomechanical cell signaling, and how these interactions can be assessed through imaging and 3D models.

## The Lung Extracellular Matrix

The term “matrisome” has been introduced to describe the more than 300 core ECM proteins and associated ECM modifying molecules, secreted factors, ECM-affiliated proteins, and ECM regulators in mammals. The lung matrisome proteins fulfil many different functions from tissue stability to biochemical-rich macromolecules governing cell behaviour [3,4,5,6,7]. To generate and maintain these complex tissue architectures, the lung ECM is made from several cell types including fibroblasts, smooth muscle cells, epithelial, endothelial and immune cells. The predominant components of the basement membrane include collagen IV, collagen V, laminins (which are also the most abundant noncollagenous component), chondroitin sulphate proteoglycans (perlecan, agrin and dystroglycan), entactin, fibronectin, fibulin I and fibulin II [8,9], whereas, the interstitial matrix comprises largely of a meshwork of elastin, fibronectin, vitronectin, tenascin, versican, decorin, fibrilar collagen I and III [10]. The biochemical properties of ECM proteins, including their insolubility, high-molecular-weight, and cross-linked structures, have traditionally made it very difficult to study the matrisome in health and disease. However, the recent use of label-free quantification by mass spectrometry is enabling phenotyping and quantification of absolute amounts of ECM within both rodent and human lungs [11,12]. A recent proteomics study on the mouse lung identified 171 core matrisome proteins and 264 matrisome-associated proteins that included secreted morphogens (Wnt, BMP/TGFb and FGF), matrix-degrading enzymes (MMPs, serpin families, S100) and extracellular vesicles [12]. The ability to resolve the lung matrisome in both health and disease offers great potential to resolve the mechanisms involved in tissue repair and remodeling.

## Extracellular Matrix Remodeling in Ageing and Chronic Lung Disease

During physiological ageing of the lungs, anatomical and functional changes include progressive loss of chest wall compliance and lung elastic recoil, as well as reduced strength in the respiratory muscles [13]. These changes are linked to a reduction of airway tissue, enlarged airspaces, and reduced alveoli surface area, which cause a rise in the residual volume and a decreased vital capacity of the lungs [14,15]. Lung ECM dysregulation has been described as one of the features of lung ageing [16]. In line with this, whole-genome profiling of lung tissue has revealed that the ECM genes of collagen III, IV and VI (COL3A1, COL4A and 1 COL6A3) are increased with ageing [17]. Glycation of different ECM fibers is important for the biomechanical properties of the lung; accumulation of (AGEs) is associated with fibrosis and tissue stiffness related to ageing [18]. It is important to note that ageing is a major risk factor for the development of chronic lung diseases such as COPD, lung cancer and IPF, and the lungs of individuals with early-onset disease show signs of rapid lung ageing [16]. Recently, de Vries et al., using whole-genome sequencing of whole lung tissue showed that the lung ECM-receptor interaction pathway was the most significantly enriched amongst genes that decrease with age and that these genes were significantly enriched in lung tissue from COPD patients, indicating accelerated lung-ageing in COPD [19]. In support of this finding, it has been shown that decellularized lung ECM scaffolds from aged mice are unable to support the repopulation of lung cells, recapitulating the findings of emphysematous lung ECM scaffolds [20]. Several studies have assessed the composition of the ECM in chronic lung diseases, such as asthma, COPD, and IPF, by studying biopsy samples, resected tissues, autopsy samples or cells isolated from the lungs [21]. Below we describe the hallmark features of ECM remodeling in the lung of patients with asthma, COPD and IPF.

## Asthma

Asthma affects an estimated 262 million people and caused over 461,000 deaths in 2019 [22]. The disease is characterized by chronic airway inflammation (which forms the target of all asthma therapeutics) and airway remodeling that involves all tissues of the airway wall, for which there are no treatments [23]. Airway remodeling was first described in cases of fatal asthma by Huber and Koessler [24]. Since then, features of airway remodeling have been documented for all stages of asthma severity and have been linked to reduced lung function, airways hyperresponsiveness, and greater use of asthma medications [23,25,26]. Abnormal thickening of the basement membrane with increased deposition of ECM within the lamina reticularis is one of the hallmarks of airway remodeling in asthma and is observed in children and adults with mild to severe and fatal asthma [27,28,29,30]. The lamina proporia of large and small conducting airways in children and adults with asthma has increased deposition of ECM, which was more recently shown to be primarily composed of disorganized fibrillar collagen [23]. While there is no increase in ECM surrounding airway smooth muscle (ASM), the increased proportion of fibronectin, elastin fibers and matrix metalloproteinase (MMP)-9 and MMP-12 [31] are related to disease severity (bronchoconstriction and bronchodilation) [32]. The ECM in the bronchial and, more recently, distal pulmonary vasculature, has also been shown to involve the deposition of disorganized fibrillar collagen in children and adults with mild and fatal asthma [33,34,35,36,37]. It was previously proposed that chronic inflammation involving mast cells, eosinophils, neutrophils, and CD4^+^ T cells lead to a chronic cycle of injury resulting in airway remodeling over the lifetime of an individual with asthma [38,39,40]. However, recent studies have now shown that airway remodeling is not present at birth [41], but features of remodeling are present by the age of 2–4 years [33,42], often before atopic inflammation is observed or a clinical diagnosis of asthma is made [43]. ECM remodeling thus affects both basement membranes and interstitial matrix in multiple lung anatomical niches in patients with asthma.

## Chronic Obstructive Pulmonary Disease

COPD is one of the most common lung diseases worldwide, and the third leading cause of death [44] due to exposure of tobacco smoke and/or environmental pollutants [45]. COPD is characterized by a progressive loss of lung function with airflow obstruction that is not fully reversible with bronchodilators [45]. Airflow obstruction in COPD is caused by a combination of factors, including pulmonary inflammation associated with bronchitis and mucus hypersecretion, small airway disease and lung emphysema in varying combinations and severities [46]. Unfortunately, no current drug treatments modify the course of the disease.

In COPD, several studies have shown a decrease in the protein expression and volume fraction of elastin in both the conducting airways and the parenchyma of patients with mild, moderate and severe COPD [47,48,49]. Further, Deslee et al. [50] showed that elastin fibers within alveolar walls of patients with severe COPD are less densely packed and loose compared to healthy subjects. However, Vlahovic et al. [47], using electron microscopy, showed that in remaining alveolar walls in regions of emphysema there is an increased volume fraction of collagen and elastin, indicating that extensive synthesis and remodeling of the ECM may occur at sites of injury [51]. Loss and disorganization of elastin within the parenchyma and small airways has thus been proposed to lead to loss of elastic recoil and airway-parenchyma uncoupling leading to air trapping in patients with COPD [52].

In terms of proteoglycans, biglycan and decorin have also been shown to decline in the small airways and the parenchyma of patients with severe COPD [53,54]. In contrast, the levels of the proteoglycan versican, which can inhibit synthesis and generation of elastic fibers, is increased in the parenchyma of patients with mild and moderate COPD [49]. Versican production by fibroblasts derived from COPD patients has also been shown to be increased compared to fibroblasts derived from nonsmokers [55]. However, the levels of versican do not appear to change in the conducting airways [56].

The glycoproteins tenascin and fibronectin have been shown to be increased in the airways of mild to moderate COPD patients, and inversely correlated with lung function [56,57,58]. In the parenchyma, no change in tenascin and fibronectin expression was found [56], although fibronectin gene expression may be reduced in severe COPD [59].

An increase in total fibrillar collagen expression in the airway and parenchyma of patients with mild, moderate and severe COPD has been reported [48]; however, the precise contribution of individual collagen subtypes to this change is unclear. In several studies, the increase in fibrillar collagen has been attributed to an increase in collagen I in the airways and parenchyma of patients with mild, moderate and severe COPD [48,56,60], whereas the expression of collagen III in patients with mild to moderate COPD was reported to be the same as controls [48,61]. In summary, ECM remodeling in COPD affects both basement membranes and interstitial matrix in multiple lung anatomical niches, and is associated with disease progression.

## Idiopathic Pulmonary Fibrosis

IPF is the most common and progressive type of idiopathic interstitial pneumonia that primarily affects older adults with a median survival of 3–5 years [62,63,64,65,66], despite two recently approved antifibrotic therapeutic agents [65,66,67]. The pathology of IPF is characterized by heterogeneous and progressive subpelural fibrosis, fibrotic foci (accumulations of myofibroblasts adjacent to areas of apoptotic or hyperplastic alveolar epithelial cells) and honeycomb cysts [67,68,69,70]. More recently, the early pathology of IPF was shown to involve the loss of small airways [71]. These pathological alterations within the tissue lead to a restrictive pattern of lung function with lower volumes on spirometry and reduced gas transfer [72].

Activated fibroblasts and myofibroblasts in fibroblastic foci are believed to be the major producers of the ECM. Several studies have shown that collagen III is predominately deposited in early IPF, whereas more collagen I deposition is present in late-stage IPF [73,74]. Within fibroblastic foci, increased expression of type I procollagen, versican, hyaluronan and tenascin-C, and decreased expression of decorin and biglycan, have been reported, indicating active ECM synthesis and remodeling [75,76]. In addition to collagen deposition, an increase in elastic fibers has been observed in IPF tissue and to correlate with worse outcomes and prognosis [77]. While elastic fibers are essential for the normal elastic recoil of the lung, an increase in elastic fibers also leads to increased stiffness of the lungs, resulting in an increased effort of breathing during early inspiration [77].

In IPF, myofibroblasts have been shown to be resistant to apoptosis and to produce increased amounts of ECM [78]. The trigger for abnormal activation and resistance to apoptosis in myofibroblasts derived from IPF patients has been suggested to be linked to an abnormal activation of the alveolar epithelium leading to increased release of growth factors such as TGF-β [70]. In addition, the stiffness of the ECM in IPF has been shown to cause an accumulation of integrins and focal adhesions that initiate mechanotransduction pathways (Rho kinase (ROCK) and focal adhesion kinase (FAK)), leading to increased myosin-II activity and actomyosin contractility that causes fibroblast to myofibroblast differentiation and the overexpression of ECM proteins such as collagen 1 [79]. Most recently, Parker et al., demonstrated that decellularized lung matrix from IPF patients had a greater impact on gene expression and protein translation, including ECM expression, irrespective of whether they were seeded with fibroblasts from controls or IPF patients [80].

The structure and the topography of the lung ECM is highly affected by cross-linking of ECM fibers by enzymes such as the lysyl oxidase (LOX) family and various transglutaminases (TGs) [81]. LOX is essential for the crosslinking of collagen and elastin fibers, while TGs are important for crosslinking different ECM proteins to create proteolysis-resistant fibers [21]. In IPF, it has been shown that increased levels of LOX-like 2 (LOXL2) is highly associated with disease progression, and the increased activity of LOX and TG enzyme families are associated with pathological crosslinking of ECM fibers and ECM stiffening [81,82]. In summary, ECM remodeling in IPF affects the interstitial matrix within the conducting and respiratory airways and is an important predictor of disease progression.

In this special issue, Blokland et al., report on the ECM deposited by IPF lung fibroblasts and its effect on the cellular environment, particularly cellular senescence. Tam et al., report on report of the potential of Hedgehog signaling as a therapeutic target in airway remodeling and inflammation using a murine allergic asthma model.

## Epigenetic Regulation of the Lung Extracellular Matrix

There is a dynamic biophysical and biochemical reciprocal interaction between cells and their surrounding ECM microenvironment [21]. Variations in cell phenotype determine the assembly and composition of ECM proteins resulting in different tissue morphologies and functions [83]. Alterations in the lung ECM environment result from the synthesis, degradation or altered organization of ECM components [84]. To initiate ECM production, cells relay signals via the membrane and cytoskeleton by recruiting factors such as cytokines, adhesion molecules and growth factors to activate gene transcription of ECM components directly, or the production of ECM remodeling enzymes (MMPs and A disintegrin and metalloproteinase with thrombospondin motifs (ADAMTS)) [85]. A number of control mechanisms are required to ensure that the expression and function of the ECM and ECM-modifying enzymes remains in balance [84]. It is now understood that the control of gene expression in eukaryotic organisms is controlled not only by the activity of transcription factors but also via epigenetic modification through DNA methylation, histone modification, ubiquitination modification, and noncoding RNA regulation [86,87]. In this special issue, Heijink and Brandsma report on the regulation of fibroblast-epithelium cross-talk by miRNAs and how this mechanism is altered in COPD. Rajasekar et al., report on the regulation of fibroblast phenotypes by DNA methylation and their contribution to lung fibrosis. Lastly, Usman et al., provide a review on the roles of miRNAs in ECM repair and chronic fibrotic lung diseases.

## Topography of the Lung Extracellular Matrix

The lung ECM is a nonlinear elastic material that becomes stiffer when stretched [88,89,90], but it is also a viscoelastic material that relaxes or creeps under a constant deformation or load, respectively [10]. The organization and topography of the ECM environment have fundamental effects on cell behaviour [91,92,93]. The topography of the ECM can be post-translationally modified by oxidation, citrullination, glycosylation, glycation and transglutamination, as well as enzymatic and chemical crosslinking of different fibers. For example, collagen-elastin intermolecular crosslinking by lysyl oxidase (LOX) and lysyl hydroxylases leads to an increase in the matrix stiffness and tensile strength, which can profoundly change cellular behaviours [94,95]. Cells can also manipulate ECM tomography through traction of ECM proteins by binding of cell receptors and anchorage with the cell cytoskeleton. Such traction interferes with the cross-linking bonds between ECM proteins and the cell’s actin cytoskeleton, leading to modification of cellular signaling and gene expression. For example, stretching of fibronectin by cellular traction force increases its binding force to integrin receptors, other fibronectin dimers and collagen [96]. This, in turn, increases the size, density and rigidity of fibronectin fibres and causes pleiotropic changes in cell growth, differentiation and migration, often influencing the progression of fibrosis in cells [96]. In this special issue, Poole et al., review recent nonlinear optical imaging techniques that can be used to assess the biomechanical properties of the ECM within lung tissues. Park et al., report on how the mechanobiology of the airway epithelium and how its influence on the ECM environment can influence airway remodeling in asthma.

## Modelling the Lung Extracellular Matrix

While two-dimensional (2D) models of cells grown on tissue culture plastic are simplistic in design, the stiffness of cell culture plastic ranges from 2 to 4 GPa, whereas normal lung tissue varies between 0.44 and 7.5 kPa, depending on the region measured, and 16.52 kPa in fibrotic human lung tissue from patients with IPF [11,97,98]. In recent years, the use of 3D tissue-engineered in vitro model systems such as air-liquid interface cultures, lung organoids, precision-cut lung slices, lung-on-a-chip models, ECM gels, and coculture systems, as well as animal studies, have been used to model the unique spatial geometry and complex cell-cell–ECM interactions within the lung [99,100]. In addition to understanding cell-cell-ECM interactions, these complex in vitro models have the potential to aid in future therapeutic lung disease studies [99,100]. As complex models are developed, the need for complex ECM scaffolds to enable cell growth and differentiation has become more evident. In this issue Dabaghi et al., report on a protocol for decellularized human lung bioink generation amenable to 2D and 3D lung cell culture. In addition, Bennet et al., provide a detailed review on the most recent lung-on-a-chip technologies used to study the lung ECM in health and disease.

## Conclusions

The pathobiology of many chronic lung diseases is associated with changes in the composition, content, and structural topography of the ECM environment. Alterations in the lung ECM are driven by synthesis, degradation and changes in topography by multiple cell types in the lung but, most importantly, dysregulation of the ECM provides a positive feedback loop to drive fibrosis progression. As highlighted in this special issue, the development of complex models and screening of human tissues is essential to understand how ECM regulation is abnormal in lung disease and how it can be targeted therapeutically. Corticosteroids constitute the mainstay therapy for managing chronic inflammatory diseases such as asthma, COPD and IPF; however, the effectiveness of steroids on ECM remodeling is controversial. A two-week study of budesonide on airway structure in asthma demonstrated no change in collagen content, but it did increase the content of the PGs versican and biglycan, showing some selective action [101]. Similarly, a 30-month study of fluticasone in patients with COPD demonstrated increased airway collagen III and versican compared with patients receiving placebo [102]. Together, the data indicate that steroids may contribute to the repair or stabilization of lung tissue structures; however, it is clear that long term use does not reverse the disease process. Targeted treatments for ECM remodeling are required as each disease pathology varies greatly in its structural niche and ECM composition; for example, ECM deposition in the interstitial matrix of airways and ECM loss in the interstitial matrix of the parenchyma in the COPD lung. The approval of pirfenidone [62] and nintedanib [63] for targeting the different pathways involved in lung fibrosis in IPF, provide excellent examples of how treatments targeted at ECM remodeling are important [103].

The investigation of the reciprocal interactions between cell-ECM remodeling in lung pathologies provides an urgently needed opportunity to identify therapies to reduce disease mortality. As highlighted in this editorial, pathological ECM remodeling occurs during childhood in asthma, and early in the disease process in COPD and IPF before small airway loss, emphysematous tissue destruction and tissue fibrosis. It is, therefore, important that studies focus on this specific time window during the early progression of lung disease. Indeed, damaged, and disorganized tissue repair may have a more reasonable chance to be targeted therapeutically, as it is physically intact tissue that can be remodeled and repaired. The goal of this special issue is to identify research focused on the disease-specific changes in cellular behaviours that are driven by the ECM and, in turn, drive fibrosis. The reviews and research articles assess the contribution of different cytokines & growth factor signaling pathways, epigenetic regulation, and biomechanical signaling involved in cell-ECM repair responses. We believe this is an important area of research that will drive new approaches to modify lung ECM remodeling and the outcomes of many chronic lung diseases.
